# A Short-Duration Gonadotropin-Releasing Hormone Stimulation Test for the Diagnosis of Central Precocious Puberty

**DOI:** 10.3390/medicina60010024

**Published:** 2023-12-22

**Authors:** Maria Chiara Pellegrin, Chiara Marzin, Lorenzo Monasta, Gianluca Tamaro, Viviana Vidonis, Giada Vittori, Elena Faleschini, Egidio Barbi, Gianluca Tornese

**Affiliations:** 1 Institute for Maternal and Child Health IRCCS Burlo Garofolo, 34129 Trieste, Italy; 2 Department of Medicine, Surgery and Health Sciences, University of Trieste, 34129 Trieste, Italy

**Keywords:** precocious puberty, GnRH stimulation test, LHRH stimulation test

## Abstract

*Background and Objectives*: The gonadotropin-releasing hormone (GnRH) stimulation test is the gold standard method for diagnosing central precocious puberty (CPP), although it requires multiple blood samplings over 120 min. This study aimed to evaluate if a shorter test may have an equivalent diagnostic accuracy. *Materials and Methods*: We retrospectively reviewed the GnRH tests of 188 consecutive pediatric patients (169 females) referred for signs of early pubertal development. The diagnostic accuracy of the hormonal levels was evaluated at different time points (15, 0, 60, 90, and 120 min after the GnRH stimulus). *Results:* A diagnosis of CPP was made in 130 cases (69%), with 111 (85%) being female. Sensitivity and specificity ratings higher than 99% for the diagnosis of CPP were achieved for LH levels ≥4.7 mU/mL at 30 and 60 min after the stimulus (area under the ROC curve (AUC) = 1), with no further increase in the diagnostic accuracy in the remaining time points. No sex differences in diagnostic accuracy were found. The LH/FSH ratio at 30 min showed a sensitivity of 94.9%, with an AUC of 0.997 and a value ≥0.76. *Conclusions:* A short-duration GnRH test of 60 min provided optimal results for the diagnosis of CPP. Extending the test for an extra hour is therefore unnecessary and inadvisable.

## 1. Introduction

Precocious puberty is defined as the onset of secondary sexual characteristics (Tanner Stage 2) before 8 years of age in girls (development of breast buds under the areola, also known as thelarche) and 9 years in boys (testicular volume ≥ 4 mL) [1]. This condition may affect up to 1 in 5000–10,000 children among Caucasians with a female predominance (approximately 10:1), resulting in short stature and social and emotional distress [2]. Suspicion of precocious puberty is one of the most frequent causes of pediatric endocrine referral [3].

Precocious puberty caused by central activation of the hypothalamic-pituitary-gonadal axis is classified as gonadotropin-dependent, also called true or central precocious puberty (CPP). Specific genetic alterations (*MKRN3*, *KISS1*, *KISS1R*, *DLK1*, *GPR54*, and also other syndromes), central nervous system lesions (e.g., hypothalamic hamartoma) and social and environmental stressors (e.g., adoption or endocrine disruptors) are major drivers of CPP, although the majority of patients with CPP have no identified etiology and are labeled as idiopathic CPP.

In CPP, the levels of gonadotropins, luteinizing hormone (LH), follicle-stimulating hormone (FSH) and sex steroids are elevated earlier than normal. However, a single blood sample may not capture the dynamic changes in hormone levels that occur over time. The pulsatile release of gonadotropin-releasing hormone (GnRH) and subsequent fluctuations in LH and FSH levels need to be evaluated to confirm the diagnosis accurately. The GnRH stimulation test is a mainstay in CPP diagnosis since it may reveal premature activation of the hypothalamic-pituitary-gonadal axis in patients with clinical signs of precocious puberty [4,5]. The GnRH stimulation test helps evaluate how the pituitary gland responds to the administration of synthetic GnRH and confirms whether puberty started earlier but in the right place.

Over the last few decades, alternative methods, such as measuring the basal LH and FSH values or the subcutaneous GnRH analog test with a single sample, have been proposed. However, they do not show accuracy equal to or better than that of the GnRH test [6,7,8]. For example, LH levels initially increase overnight. Therefore, while a high basal LH can confirm a diagnosis of CPP, a low basal LH level cannot completely rule out CPP.

A GnRH stimulation test may only be performed in a dedicated clinical investigation setting and requires several blood samplings to measure the LH and FSH levels at different time points after the stimulus [9,10]. Different timing of blood sampling has been proposed [11]. Traditionally, GnRH is administered via an intravenous bolus with 5–8 subsequent blood samplings at 15–30 min intervals. The test duration is usually between 90 and 120 min, and 15–25 mL of blood are required. Therefore, the GnRH test is often considered time- and resource-consuming and uncomfortable for patients [12]. Some evidence suggests that the diagnosis of CPP might even be possible by reducing the duration of the test, since LH values greater than 5 mU/mL are already reached after 30–45 min [13,14]. However, the best time to measure LH after GnRH stimulation is still unclear, and different cut-off values have been proposed. Also, the role of the LH/FSH ratio is not clearly defined. Evidence is conflicting, and no study from a European country has been published yet on this issue.

This study aims to determine whether a shorter duration for the GnRH test is adequate for diagnosing CPP in both sexes to stop the stimulation test earlier, with numerous advantages for patients and hospital staff.

## 2. Materials and Methods

### 2.1. Population

We retrospectively reviewed the medical records of 188 consecutive pediatric patients (19 males and 169 females) referred to the Endocrinology Unit of the Institute for Maternal and Child Health IRCCS Burlo Garofolo in Trieste, Italy from January 2004 to March 2019 for signs of early pubertal development. The indication for the GnRH stimulation test was the onset of thelarche before the age of 8 years in girls and a testicular volume ≥4 mL before the age of 9 years in boys. Data evaluation included the history of the onset and progression of pubertal signs, familiar history or adoption, auxological data (Tanner stage, height, weight, and height velocity), bone age, basal FSH and LH levels, and GnRH stimulation testing. The “G2 clinico” platform (management system specialist activities) was employed to access all patients’ data.

### 2.2. Laboratory Tests

The GnRH stimulation test was performed using an intravenous bolus of Gonadorelin (Relefact; Sanofi-Aventis, Frankfurt am Main, Germany) at a dose of 100 µg/m^2^ according to protocols as previously described [15]. An intravenous cannula was inserted in fasting patients, with blood samplings before the bolus and at 15, 30, 45, 60, 90, and 120 min after the stimulus. The LH and FSH levels were determined in all patients at each time point with an immunochemiluminometric assay (ICMA). The kits used were the FSH IRMA kit and Access hLH kit, both of which are compatible with the DxI 9000 (Beckman Coulter, Brea, CA, USA). The hormones had an intra-assay coefficient of variation below or equal to 4.05% and an inter-assay coefficient of variation below or equal to 8.2%. For the FSH hormone, the analytical sensitivity was from 0.2 IU/L for the lowest level to the highest calibrator of 80 IU/L. Instead, the LH hormone‘s lowest detectable level distinguishable from zero (Access hLH Calibrators; Beckman Coulter, Brea, CA, USA) with 95% confidence was 0.1 IU/L. According to the results for the LHRH stimulation test, CPP was defined with an LH peak ≥5 mU/mL in at least one blood sample.

Estradiol was measured among females with Access estradiol by chemiluminescence with a detection limit of 5 pg/mL. Testosterone levels were analyzed in the male patients. The Access testosterone test was used, employing a competing enzyme immunoassay where the limit of detection was 0.1 ng/mL. Estradiol values greater than 10 pg/mL and testosterone levels above 0.25 ng/mL were considered pubertal [16].

### 2.3. Clinical and Radiological Assessment

The height was measured using a wall-mounted Harpenden stadiometer to the nearest 0.1 cm, and the weight was measured using an electric digital scale to the nearest 0.1 kg. The height, weight, and BMI were considered according to Italian growth charts and expressed as standard deviation scores (SDSs) [17]. The growth rate was considered according to the Tanner charts [18] and counted as accelerated when greater than 1 SDS. The height, weight, BMI, and height velocity SDSs were determined by employing Growth Calculator software version 3.0 (Weboriented, Turin, Italy).

Bone age was evaluated by two pediatric endocrinologists using the method of Greulich and Pyle [19]. Advanced bone age was defined when the difference with the chronological age was greater than 1.5 years.

Transabdominal 2D gray-scale pelvic ultrasound (US) was performed using a Voluson E10 (General Electric Healthcare GE, Zipf, Austria), with a 1–5 MHz transabdominal transducer with a full bladder. The uterus was visualized along the longitudinal, transverse, and anteroposterior axes to calculate the volume and the ratio between the height of the body and the uterine cervix. The endometrial echogenicity and thickness were evaluated and reported. The longitudinal, transverse, and anteroposterior axes of the ovaries were calculated to determine their volume. The pelvic ultrasound was defined as pubertal when the uterine length was greater than or equal to 3.4 cm, the ovarian volume was greater than or equal to 2 mL, or in case of a measurable endometrial rim [1].

### 2.4. Ethical Statement

This study was approved by the Institutional Review Committee of IRCCS “Burlo Garofolo” (approval number: RC 13/17 Line 2). Ethical committee approval was not requested since General Authorization to Process Personal Data for Scientific Research Purposes (authorization no. 9/2014) declared that retrospective archive studies that use ID codes, preventing the data from being traced back directly to the data subject, do not need ethics approval [20]. According to the research institute’s policy, informed consent was signed by the parents at the first visit, in which they agreed that “clinical data may be used for clinical research purposes, epidemiology, study of pathologies and training, with the objective of improving knowledge, care and prevention”.

### 2.5. Statistical Analysis

Continuous variables were assessed for normality with the Shapiro–Wilk test. Values were mostly reported as medians and interquartile ranges. The Mann–Whitney U test was used to compare medians. The Student’s *t* test was used to compare the means for normally distributed variables. Data were then visually represented using ROC curves and graphs. To compare the ROC curves obtained, we performed a nonparametric analysis of the ROC curves using bootstrapping. A *p* value < 0.05 was considered statistically significant.

## 3. Results

The GnRH test was performed with 188 patients (169 females), and a diagnosis of CPP was made in 130 cases (69%, with 111 females and 19 males). The test was positive in 66% of females, while CPP was diagnosed in all male patients. Thirteen children (10%) were adopted and were of non-Caucasian ethnicity. Sixteen (12%) reported a family history of CPP (14 females and 2 males). The clinical features and laboratory parameters of the study population are shown in Table 1.

### 3.1. GnRH Test in Females

**Figure 1:**
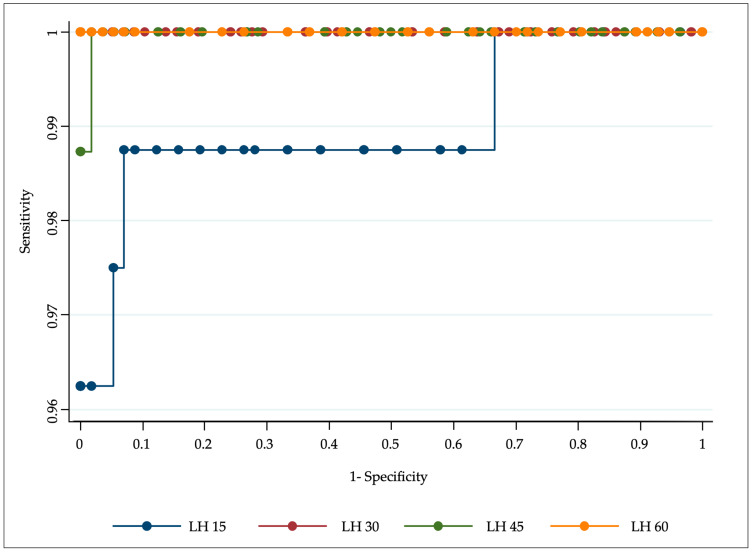
Receiver operating characteristic curves for central precocious puberty diagnosis with LH levels at 15, 30, 45, and 60 min.

In the females, a sensitivity of 96.2% and specificity of 100% for the diagnosis of CPP were reached after 15 min from the GnRH stimulus, considering as a cut-off an LH peak ≥3.8 mU/mL. The area under the curve (AUC) was 0.99 (Figure 1).

At 30 min, considering an LH peak ≥4.8 mU/mL as the cut-off, we found a sensitivity and specificity of 100%, with an AUC equal to one (Figure 1). When maintaining the standard cut-off of 5 mU/mL, the sensitivity was 99%.

At 45 min, the sensitivity was 98.7% and the specificity 100% when considering an LH surge greater than 5 mU/mL.

At 60 min, a diagnosis of CPP could be made with a sensitivity and specificity of 100%, taking as a limit an LH peak ≥4.7 mU/mL.

By analyzing the cumulative frequency of patients with LH levels higher than 5 mU/mL (Table 2), it can be observed that 95.5% of the patients with CPP already reached an LH value above the cut-off of 5 mU/mL after 30 min from the start of the test. Almost all of the patients reached this value after 45 min. Similarly, when keeping an LH value greater than 3.3 mU/mL as the cut-off, 96.4% of the patients reached this value after 30 min from the start of the test, and almost all of them reached it after 45 min (Table 2).

**Figure 2:**
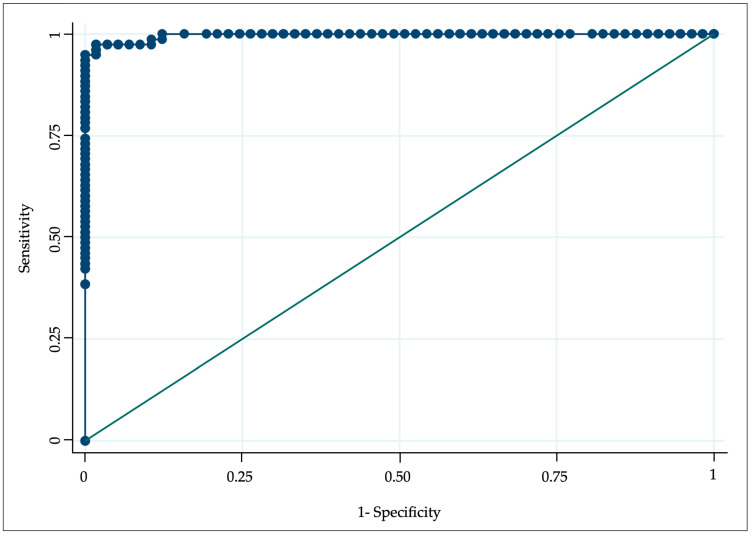
Receiver operating characteristic curves for central precocious puberty diagnosis with LH/FSH ratio at 30 min.

An LH/FSH ratio greater than or equal to 0.8 at 15 min showed a sensitivity of 89.5% with an AUC of 0.993, while at 30 min, it reached the best performance, with a sensitivity of 94.9% for a ratio greater than or equal to 0.76 and an AUC of 0.997 (Figure 2).

In subsequent measurements, the sensitivity and specificity for the LH/FSH ratio decreased slightly. After 45 min, an LH/FSH ratio greater than or equal to 0.80 had a sensitivity of 92.3%, with an AUC of 0.996. A similar sensitivity (92.2%) was provided at 60 min by a ratio greater than or equal to 0.73 and an AUC of 0.995.

The baseline estradiol values did not have predictivity for the diagnosis of CPP.

**Figure 3:**
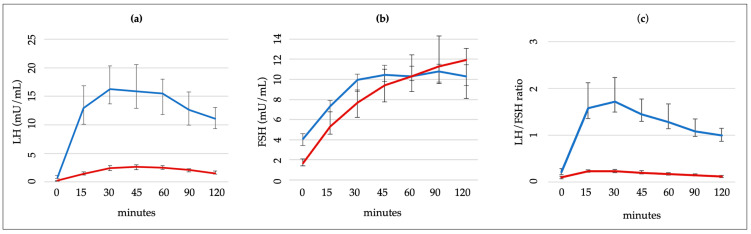
LH (**a**) and FSH (**b**) levels and LH/SH ratio (**c**) after the stimulus. Blue lines = females with central precocious puberty; red lines = females without central precocious puberty.

Figure 3 shows the LH and FSH levels and LH/FSH ratio among females with and without CPP at different times after the GnRH stimulus.

### 3.2. GnRH Test in Males

The peak in LH levels was reached in 13 male patients after 30 min and in 6 patients after 45 min. A diagnostic LH value greater than 5 mU/mL was achieved by 84.2% of the patients at 30 min (Table 3).

When using an LH value greater than 3.3 mU/mL as the cut-off, the results did not vary significantly, with 84.2% of patients reaching this limit after 30 min.

### 3.3. Gender Differences

**Figure 4:**
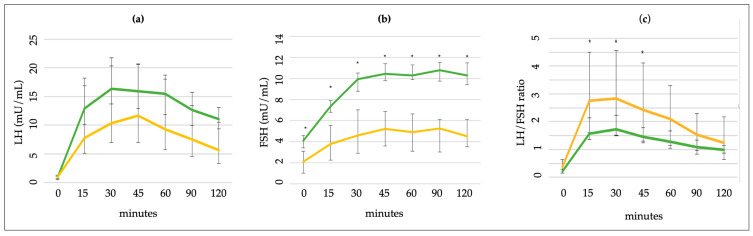
Comparison of (**a**) LH and (**b**) FSH levels and (**c**) LH/FSH ratio in males and females after GnRH stimulus. * *p* < 0.05 Mann–Whitney U test. Green lines = females; yellow lines = males.

When comparing the LH levels reached during the GnRH test in males and females with CPP, no significant difference was observed at any time point (Figure 4). The FSH levels after the stimulus were significantly higher in females than in males (*p* < 0.0001 at every time point (Figure 4)). A significantly higher LH/FSH ratio was observed in males after 15, 30, and 45 min (Figure 4).

## 4. Discussion

The suspicion of precocious puberty is one of the main reasons for referral to a pediatric endocrinologist [2]. The number of patients diagnosed with CPP is increasing [21], and a spike in new diagnoses was noticed during the COVID-19 pandemic [22,23]. The gold standard for diagnosing CPP is the GnRH test, although it has a considerable economic cost, requires between 90 and 120 min, and may cause significant distress in the patients.

We showed that a short-duration GnRH test with only two samples at 30 and 60 min provides optimal results for the diagnosis of CPP. Sensitivity and specificity values higher than 99% for the diagnosis of CPP were achieved in females with LH levels ≥4.7 mU/mL at 30 and 60 min after the stimulus (AUC equal to one), with no further increase in the diagnostic accuracy in the remaining time points. By analyzing the cumulative frequency of patients with LH levels ≥3.3 or ≥5 mU/mL, only at 120 min did the totality of the female patients with CPP reach these values. while all males had a value greater than both cut-offs from 45 min onward. This is why we suggest that obtaining only two samples at 30 and 60 min provides 100% sensitivity and specificity for diagnosing CPP in both sexes.

To our knowledge, this study represents the first evidence of the diagnostic accuracy of the GnRH test for CPP in Europe, and our data confirmed that samples of LH at 30 and 60 min after stimulus are also sufficient to provide optimal accuracy in males. Our findings agree with the data of Kim et al. [14]. In their study conducted in a Korean population, values of LH measured from a single blood sample obtained 45 min after GnRH stimulation were considered adequate for the diagnosis of CPP. However, the sample size in this study was smaller and did not include males. Also, in the test with subcutaneous GnRH analogs, in which a measurement at least 180 min after injection is usually required [24,25], samples at 30 min [26] or 60 min [27] were found to be a reliable alternative for diagnosing CPP.

Regardless of which test is used, an LH/FSH ratio >0.66 is also considered pubertal [28]. In our study, the peak value of the LH/FSH ratio was higher in pubertal girls (0.61 vs. 0.27, *p* < 0.001), was reached in the first hour from the stimulus, and at 30 min showed a sensitivity of 94.9% with an AUC of 0.997 and a value ≥0.76, representing an additional value in the diagnosis of CPP. We also found a significantly higher LH/FSH ratio in males (at 15, 30, and 45 min) due to the higher FSH levels in females (11.3 vs. 6.3 mU/mL), which is known in the early stages of puberty [13].

In our study, females with CPP also had a significantly higher LH level at baseline compared with those without a pubertal response to the GnRH test (0.8 vs. 0.2 mU/mL, *p* < 0.0001). Many previous studies have already evaluated different basal LH thresholds for CPP screening. However, there are disagreements regarding the appropriate cut-off values and mixed results for the sensitivity and specificity [29,30,31,32,33]. In another study from our group, by using a threshold of a basal LH ≥1 mU/mL, we already demonstrated that 31% of the patients could have spared a GnRH test and that the rate of false-negative CPP was 0% [34]. For this reason, we did not further evaluate this point in the present study. However, we advocate for GnRH testing mainly when basal LH levels are nondiagnostic [2].

Although CPP is almost invariably associated with high increased growth [1], in our cohort, the height velocity was not significantly higher in CPP (2.4 vs. 1.8 SDS, *p* = 0.456). As already reported, height velocity may not help in discriminating CPP from premature thelarche (PT) or the thelarche variant [15], and studies have shown that the growth rate (>7 cm/year) is also a strong predictor for PPC, underlining an overlap in growth velocity between girls with PT and CPP [34].

Despite the conventional association of CPP with a pronounced increase in growth [1], our study’s analysis of the height velocity in the cohort did not yield a statistically significant difference between the CPP and no CPP groups (2.4 vs. 1.8 SDS, *p* = 0.456). This intriguing discrepancy prompts a closer examination of the nuanced relationship between CPP and growth patterns, challenging prevailing expectations. It is noteworthy that the lack of a discernible elevation in height velocity within the CPP cohort contradicts the findings from previous research, adding a layer of complexity to our understanding of the interplay between puberty and growth dynamics. Consistent with the existing literature [15], our study suggests that the height velocity alone may not serve as a reliable discriminator between CPP and premature thelarche (PT). This aligns with the recognition that the growth patterns in girls with PT and CPP may exhibit overlapping characteristics, challenging the utility of height velocity as a singular diagnostic criterion. The nuanced relationship between the growth rate and pubertal development is further emphasized by studies highlighting a growth rate threshold (>7 cm/year) as a robust predictor for precocious puberty, potentially indicating a shared spectrum of growth velocities between different pubertal conditions [35]. The intricate interplay between pubertal development and growth necessitates a holistic and nuanced approach to diagnostic criteria, recognizing the potential overlap and variability inherent in the growth trajectories of individuals experiencing different forms of pubertal onset.

Our study also failed to reveal any statistically significant difference in the estradiol levels between girls with or without CPP, registering values of 16.8 pg/mL and 21.0 pg/mL, respectively. This aligns with the findings of precedent studies that also reported nonsignificant variations in estradiol levels under comparable circumstances [36]. Interestingly, our observations stand in contrast to divergent reports from other studies [34], highlighting the inherent variability in research outcomes within this domain. It is imperative to acknowledge the intricate nature of estradiol dynamics in the context of puberty and CPP. While the prevailing literature suggests that a clearly elevated estradiol level is indicative of the initiation of puberty, our study reinforces the critical notion that a low estradiol concentration should not be hastily interpreted as excluding the possibility of CPP diagnosis. Intriguingly, some girls with central precocious puberty may exhibit estradiol levels that still fall within the prepubertal range [2]. This underscores the complexity of hormonal fluctuations and underscores the importance of considering a spectrum of estradiol concentrations in the diagnostic process of CPP, recognizing that not all cases will conform to a straightforward pattern of hormonal elevation. Consequently, a nuanced interpretation of estradiol levels becomes essential in refining the diagnostic criteria for CPP, acknowledging the inherent variability and individualized nature of hormonal dynamics in the early stages of pubertal development.

Some limitations of this study should be considered. Firstly, the retrospective nature of the investigation introduces inherent constraints as it relies on historical data, potentially limiting the depth and scope of the analysis. Additionally, the study’s data emanate from a singular center, diminishing the external validity or generalizability of the results to broader populations. The restricted focus on a single institution may not capture the full spectrum of diversity present in different healthcare settings. Moreover, the study encounters limitations specific to the male sample, notably the modest sample size, coupled with the exclusive diagnosis of CCP in all male patients, precluding a meaningful comparison with a prepubertal condition, as was successfully conducted for the female cohort.

## 5. Conclusions

We can conclude that the GnRH test suspended after 30 min has a rather high sensitivity (99.03%) and specificity in the diagnosis of CPP in females, taking the value of 5 mU/mL as the cut-off. Two samples obtained after 30 and 60 min provided 100% sensitivity and specificity for diagnosing CPP in both sexes. Extending the test for an extra hour is therefore unnecessary and inadvisable.

Stopping the test early is undoubtedly an advantage not only in terms of costs but also time saved for patients, parents, and healthcare staff. For children, this indeed equates to less psycho-physical stress to which they will be subjected.

## Figures and Tables

**Table 1:** Clinical and laboratory parameters in males and females with or without pubertal response to GnRH stimulus. Data expressed as median [interquartile range]. *p* = Mann–Whitney U test.

	All Patients	Females	Females with Pubertal Response (N = 111)	Females with Prepubertal Response (N = 58)	*p* Value (Pubertal vs. Prepubertal Females)	Males with Pubertal Response	*p* Value (Pubertal Females vs. Males)	*p* Value (Females vs. Males)
Number	188	169	111	58	/	19		/
Age (years)	8.1 [7.6–8.6]	8.0 [7.5–8.5]	8.3 [7.8–8.7]	7.7 [6.7–8.0]	<0.0001	9.0 [8.3–9.4]	<0.0001	<0.0001
Age at puberty (years)	7.0 [7.0–7.9]	7.5 [6.9–7.8]	7.5 [7.0–7.9]	7.0 [6.3–7.5]	0.011	8.5 [7.9–8.9]	<0.0001	<0.0001
Tanner stage (%) (2/3/4)	69/30/1	69/30/1	53/46/1	93/7/0	/	69/25/6	/	/
Bone age (years)	10.0 [8.8–11.0]	10.0 [8.8–11.0]	10.0 [8.8–11.0]	8.8 [7.8–8.8]	<0.001	10.0 [9.0–12.0]	0.496	0.026
Age and bone age difference (years)	1.6 [0.8–2.6]	1.6 [0.8–2.6]	2.1 [1.3–2.8]	0.8 [0.1–1.5]	<0.001	1.6 [0.7–2.9]	0.259	0.889
Height velocity (SDS)	1.9 [0.8–4.4]	1.9 [0.8–4.5]	2.4 [0.8–4.6]	1.8 [0.7–3.3]	0.456	1.9 [−0.2–3.7]	0.634	0.732
Basal LH (mU/mL)	0.5 [0.2–1.2]	0.3 [0.2–1.0]	0.8 [0.3–2.0]	0.2 [0.1–0.2]	<0.001	1.1 [0.5–1.2]	0.656	0.139
Basal FSH (mU/mL)	3.0 [1.8–4.8]	2.9 [1.6–4.6]	4.1 [2.8–5.4]	1.8 [1.3–2.7]	<0.001	3.0 [1.0–3.7]	<0.0001	0.018
Basal LH/FSH ratio	0.16 [0.08–0.34]	0.15 [0.07–0.32]	0.21 [0.10–0.44]	0.10 [0.06–0.16]	<0.001	0.37 [0.13–0.66]	0.03	0.001
Peak LH (mU/mL)	10.2 [4.5–21.5]	5.8 [2.8–7.5]	17.4 [10.2–28.6]	2.7 [1.7–3.3]	<0.001	11.6 [7.1–22.8]	0.058	0.241
Peak FSH (mU/mL)	10.8 [8.3–14.3]	11.3 [8.9–15.1]	10.9 [9.0–13.4]	12.3 [8.1–18.7]	0.236	6.3 [3.7–8.5]	<0.0001	<0.0001
Peak LH/FSH ratio	0.41 [0.25–1.24]	0.39 [0.25–1.17]	0.61 [0.29–1.57]	0.27 [021–0.38]	<0.001	0.64 [0.31–1.40]	0.705	0.320
Estradiol (pg/mL)	/	18.5 [10.4–34.1]	16.8 [10.9–34.6]	21.0 [5.0–33.8]	0.497	/	/	/
Testosterone (ng/mL)	/	/	/	/	/	0.19 [0.09–1.01]	/	/

**Table 2:** Cumulative frequencies of females with LH levels greater than 5 mU/mL and 3.3 mU/mL during the GnRH test. m = minutes.

	Basal	15 m	30 m	45 m	60 m	90 m	120 m
Cumulative frequency LH > 5 mU/mL	6	72	106	110	110	110	111
%	5.4	64.9	95.5	99.1	99.1	99.1	100
Cumulative frequency LH > 3.3 mU/mL	16	81	107	110	110	110	111
%	14.4	73	96.4	99.1	99.1	99.1	100

**Table 3:** Cumulative frequencies of males with LH levels greater than 5 mU/mL and 3.3 mU/mL during the GnRH test. m = minutes.

	Basal	15 m	30 m	45 m	60 m	90 m	120 m
Cumulative frequency LH > 5 mU/mL	0	12	16	19	19	19	19
%	0	63.2	84.2	100	100	100	100
Cumulative frequency LH > 3.3 mU/mL	1	14	16	19	19	19	19
%	5.3	73.7	84.2	100	100	100	100

## Data Availability

The datasets generated during or analyzed during the current study are not publicly available but are available from the corresponding author upon reasonable request.
